# Time study on the uptake of four different beta-blockers in garden cress (*Lepidium sativum*) as a model plant

**DOI:** 10.1007/s11356-020-11610-5

**Published:** 2020-11-18

**Authors:** Franz Mlynek, Markus Himmelsbach, Wolfgang Buchberger, Christian W. Klampfl

**Affiliations:** Institute of Analytical Chemistry, Johannes Kepler University, Altenbergerstrasse 69, 4040 Linz, Austria

**Keywords:** Plant metabolism, Pharmaceuticals, Beta-blocker, Environmental analysis, Plant uptake

## Abstract

The aim of this study was to investigate the uptake of four beta-blockers by the model plant *Lepidium sativum* (garden cress) and their possible metabolization over a time period of 8 days. Therefore, cress was grown hydroponically in tap water for a week until they were matured, following irrigation with drug-containing water over the course of another 8 days. Samples were taken at days 1, 2, 4, and 8 after irrigation started. All four beta-blockers were taken up by the plants and the different octanol-water coefficients (log *P*) of the drugs have an influence on the uptake speed in the roots of the plants. The log *P* seems to have no influence on the translocation of the drugs from the root to the shoots. Furthermore, neither phase I nor phase II metabolization occurred inside the plants.

## Introduction

As water policy is a big topic in the European Union (EU), there are several directives (e.g., 2008/105/EC (European Parliament 2008), 2013/39/EU (European Parliament 2013)) with the purpose of ensuring a high water quality. In addition, the EU campaign “Water is too precious to waste” should point out the increase of water shortages and droughts across the EU in the past years. Water scarcity already affects at least 11% of the population in Europe and 17% of the EU territory respectively. Furthermore, by the year 2030, half of Europe’s river basins could be afflicted (European Comission 2020a, 2020b). In this context, one solution to face water scarcity is the reuse of treated wastewater (TWW), as already done in Malta and Cyprus, for example, where 90% and 60% respectively of the TWW is reused. Countries like Greece, Spain, and Italy only recycle between 5 and 12% of their TWW, so there is a huge potential for improvement in the usage of TWW (European Comission 2020b).

One potential field of application of TWW is for irrigation in agriculture. As might be expected, reclaimed waters employed in agriculture have to meet certain quality criteria as those presented by the European Commission in the report “Minimum quality requirements for water reuse in agricultural irrigation and aquifer recharge” (Alcalde-Sanz and Gawlik 2017). Although there are limits set in EU regulatories for microbiological and physico-chemical parameters, none is specified yet for “compounds of emerging concern (CEC).” CECs cover a wide range of different substances including pharmaceuticals and personal care products (PPCPs).

Although Paranychianakis et al. (2011) claimed that the risk coming from pesticides applied to crops is by far higher compared to CECs in irrigation water, they admit a knowledge gap, especially when the irrigation water contains a mixture of CECs. Furthermore, it is still unknown how different plants deal with multiple kinds of CECs.

So far, several studies have dealt with the determination of various PPCPs in the effluent of wastewater treatment plants (WWTPs) (Buchberger 2011; Gavrilescu et al. 2015; La Farre’ et al. 2008; Nebot et al. 2015; Petrie et al. 2013; Puckowski et al. 2016; Richardson and Ternes 2014). One important group among the classes of pharmaceuticals frequently prescribed and therefore found in almost every WWTP effluent are beta-blockers. Focusing on human metabolism, a different picture is observed for the four beta-blockers under investigation. Whereas metoprolol (MET) and propranolol (PRO) are taken up and metabolized by the human body and only a percentage below 10% is excreted via kidney unchanged and ending up in the urban wastewater, the opposite is true for atenolol (ATE) and sotalol (Godoy et al. 2015; Paterson et al. 1970; Regårdh and Johnsson 1980). According to the IQVIA market report on the development of the pharma market in Germany in 2017, beta-blockers are number four among the most widely used drugs in Germany, with about 43.7 million packages being sold (IQVIA Market report 2017). Consequently, despite the high extent of metabolization in the human body, these large amounts of daily consumed beta-blockers together with possibly illegally disposed pharmaceuticals still ensure detectable amounts of intact beta-blockers in various water bodies (Puckowski et al. 2016).

Beta-blockers, like MET and PRO, can be found in the ng L^−1^ to lower μg L^−1^ range in treated wastewater (Godoy et al. 2015) as their removal rate in WWTPs is only around 50% (Deblonde et al. 2011). Therefore, when considering the use of reclaimed wastewaters for irrigation in agriculture, contaminants like beta-blockers might be taken up by the plant roots, translocated to various plant parts, and even metabolized within the plant (Bartrons and Peñuelas 2017; Klampfl 2019).

Focusing on the interaction of beta-blockers and plants, carrots and sweet potatoes were grown in lysimeters containing different soils and were irrigated with fresh water, TWW, and spiked TWW in a study by Malchi et al. (2014). In total, 14 pharmaceutical compounds were analyzed regarding their uptake behavior, among other MET. Thereby, the latter substance could be detected in soil and, regarding plant parts, in leaves only, for both carrot and sweet potato.

In experiments conducted by Williams et al. (2015), PRO and carbamazepine (both spiked at a concentration of 1 mg kg^−1^ soil) were taken up by ryegrass (*Lolium perenne*). They investigated a possible filtering effect of three different biochars with specific properties caused by various plant materials and manufacturing processes. Therefore, they spiked soil with PRO and compared ryegrass grown in unamended soil with the plants grown in the soils amended with the three kinds of biochar. The result from this study was that PRO was taken up to a lesser extent than carbamazepine, and no difference between unamended and biochar-amended soil was observed.

Wu et al. (2013) exposed four plants (lettuce, spinach, cucumber, pepper) at two different concentration levels (0.5 and 5 g L^−1^) in hydroponic cultivation to a mix of 20 PPCPs, including ATE. The bio-concentration factor (BCF) for ATE in the plant roots was around one in the case of lettuce, between 1 and 10 for spinach and cucumber, and close to 100 for pepper. In the case of plant leafs, the BCF for ATE in lettuce was significantly below one, and between 1 and 10 for the other three plants.

In the present study, the uptake, translocation, and formation of potential drug-related metabolites of four beta-blockers, namely ATE, bisoprolol (BIS), MET, and PRO, were studied in *Lepidium sativum* (garden cress) as a model plant over a time range of 8 days. Cress was chosen as it is already fully developed after 1 week of growing, thereby keeping the time-effort for the experiments conducted relatively short. Furthermore, the possibility to employ hydroponic conditions for growing allows the simple addition of the drugs of interest to the growing medium. One major goal of this study was to gain deeper insight into the uptake mechanism of these drugs by plants, and their relation with physico-chemical parameters like log *P*.

## Experimental

### Materials and methods

The following beta-blockers used in this study were pharmaceutical formulations purchased in a local pharmacy: ATE (50 mg ATE, CAS 29122-68-7, 1A Pharma GmbH, Vienna, Austria), BIS (1.25 mg BISfumarate, CAS 104344-23-2, Sandoz GmbH, Kundl, Austria), MET (47.5 mg MET succinate, CAS 98418-47-4, Hexal Pharma GmbH, Vienna, Austria), and PRO (10 mg PRO-hydrochloride, CAS 13071-11-9, AstraZeneca GmbH, Vienna, Austria). Their physico-chemical properties can be seen in Table 1. Concentrations of the beta-blockers given within this manuscript refer to the substance, not to the salt. Diclofenac sodium salt (CAS 15307-79-6) used as internal standard (ISTD) was purchased from Sigma-Aldrich (Steinheim, Germany). The cress seeds “smooth cress, broad-leaf” (Kiepenkerl, Everswinkel, Germany) were purchased in a local garden shop.

**Table 1:** Physico-chemical properties of the four selected beta-blockers (SciFinder^n^)

Pharmaceutical	Molecular weight g mol^−1^	Water intrinsic solubility g L^−1^	pK_a_	log *P*
ATE	266.34	13	9.4	0.33
BIS	325.4	8.1	9.4	1.9
MET	267.36	11	9.4	1.6
PRO	259.34	0.5	9.5	2.9

Ultrapure water (18 MΩ cm) was obtained by a Milli-Q water purification system (Merck Millipore, Darmstadt, Germany). Acetonitrile (ACN, CAS 75-05-08, HPLC grade) and methanol (MeOH, CAS 67-56-1, HPLC grade) were supplied by VWR Chemicals (Vienna, Austria). Formic acid (FA, CAS 64-18-6, 96%) was from Sigma-Aldrich (Steinheim, Germany) and hydrochloric acid (HCl, CAS 7647-01-0, 37%) from Fisher Chemical (Vienna, Austria).

A total of 1000 mg L^−1^ stock solutions were prepared individually in MeOH. Each solution was further diluted for irrigation in tap water to a final concentration of 1 mg L^−1^. The concentration of 1 mg L^−1^ was selected as it was low enough to avoid interferences with plant growth on the one side and high enough to facilitate accurate quantitative analysis on the other side.

### Instrumentation

A 1260 Infinity II modular HPLC system (Agilent Technologies, Santa Clara, USA) was used with an Agilent InfinityLab Poroshell 120 Bonus-RP column (3.0 × 100 mm, 2.7 μm) combined with a C18 Guard column (4 × 3.0 mm, Phenomenex).

For quantification, an Agilent Technologies 6460 Triple Quadrupole mass spectrometer (QQQ-MS) equipped with a Dual Agilent Jet Stream Electrospray Ionization (Dual AJS-ESI) source was used as a detector. The Dual AJS-ESI was operated in positive mode. Nitrogen was used as drying gas and sheath gas. The temperature of drying gas and sheath gas was 275 °C, both with a flow rate of 11 L min^−1^. The nebulizer gas pressure was 45 psi, the capillary voltage was 4000 V, and the nozzle voltage was 1000 V. The fragmentor voltage was set for each compound individually as listed in Table 2 together with the precursor, quantifying, and qualifying ions as well as their respective collision energies.

**Table 2:** Retention times (RT) and MRM transitions of four beta-blockers and Diclofenac as internal standard (ISTD)

Compound name	RT (min)	Fragmentor voltage (V)	Precursor ion (m/z)	Quantifier		Qualifier	
				Product ion (m/z)	Collision energy (V)	Product ions (m/z)	Collision energy (V)
ATE	2.5	122	267.2	145.1	24	190.1; 116.1	16; 16
BIS	4.1	120	326.1	116.1	16	147.1; 204.1	20; 16
Diclofenac	6.9	100	296.0	215.0	20	250.0	15
MET	3.9	128	268.2	116.1	16	159.1; 191.1	20; 12
PRO	4.3	92	260.2	155.1	24	183.1; 116.1	12; 12

For tentative identification of possible metabolites, an Agilent Technologies 6560 DTIM QTOF-MS equipped with a Dual AJS-ESI source and a gas kit (Alternate Gas Kit, Agilent Technologies) was used as a detector, which was operated in positive mode. Nitrogen was employed as drying gas and sheath gas (both at a flow rate of 10 L min^−1^); the gas temperatures were maintained at 275 °C. The fragmentor voltage was 400 V, the capillary voltage 3500 V, the nozzle voltage 1000 V, and the nebulizer gas pressure set to 50 psi.

The mobile phase consisted of ultrapure water with 0.1% formic acid (v/v) (A) and acetonitrile with 0.1% formic acid (v/v) (B). The gradient elution was employed as follows: 0% B from 0 until 0.2 min, rising to 90% B within 5 min, hold 90% B for 2 min, and re-conditioning with 0% B for 5 min.

For the quantitative analysis, a matrix-matched calibration with the addition of an internal standard (ISTD) was done automatically by the autosampler of the HPLC system. Therefore, the following autosampler procedure was employed: needle wash (2 s); draw 10 μL of matrix (untreated cress root extract or untreated cress leaf extract); needle wash (2 s); draw 1, 5, or 10 μL of sum standard (0.1, 1, 10 mg L^−1^); needle wash (2 s); draw 2 μL of ISTD (1 mg DCF L^−1^); mix in the air (5 times); and inject.

For the quantitative analysis of the plant extracts used for the time study, the ISTD was added automatically by the autosampler of the HPLC system. The following autosampler procedure was employed: needle wash (2 s), draw 10 μL of plant extract (treated cress root extract or treated cress leaf extract), needle wash (2 s), draw 2 μL of ISTD (1 mg DCF L^−1^), mix in the air (5 times), and inject.

The matrix-matched calibration was indispensable since a significant difference in signal intensities was observed for the two different plant matrices (root/leaf). Thereby, a reduced slope was obtained for the calibration curve related to the leaf matrix.

### Germination and growing of cress

The cress seeds were germinated for 7 days in tap water on a propagator tray. Then, the watering solution was exchanged with drug-containing water. The cress plants were grown for another 8 days whereby water was added on a daily basis to compensate for evaporation. Cress plants were always grown as triplicates for each beta-blocker.

### Harvesting, extraction, and analysis of cress

**Fig. 1:**
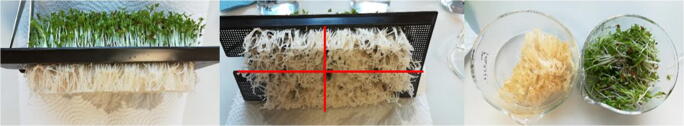
Cress plants at day 1 of the irrigation with drug-containing water. Whole plants (left), roots with the sectioning into days 1, 2, 4, and 8 (middle), and after harvest with separation into roots and leafs (right).

The propagator tray was divided into 4 sections and the plant parts were harvested after day 1, day 2, day 4, and day 8. In the process of harvesting, the roots and leafs of the cress plants were separated. The plant parts were washed twice with ultrapure water, blotted dry, and about 1.5 g roots or leafs (wet weight) were weighed in. An example for this procedure can be seen in Fig. 1.

A total of 3 mL 0.1 M HCl was added to the plant material and the mixture was homogenized with an UltraTurrax (Type TP18/10, Janke&Kunkel IKA-Labortechnik, Staufen, Germany). The slurry was centrifuged at 4000 rpm for 8 min and the supernatant was filtered with 0.45 μm Rotilabo® nylon syringe filters (Roth, Karlsruhe, Germany) into 1.5-mL HPLC glass vials (VWR, Vienna, Austria) and the samples were stored at − 80 °C until analysis.

### Data recording and evaluation

For data, recordings of Agilent MassHunter Workstation LC/MS Data Acquisition for 6400 Series Triple Quadrupole (Version 10.0 SR1) and Agilent MassHunter Optimizer (Version 10.0 SR1) were used. Quantitative analysis was done using Agilent MassHunter Quantitative Analysis for QQQ (Version 10.1).

For qualitative data evaluation, Agilent MassHunter Qualitative Analysis B.07.00 was used.

## Results and discussion

To begin with, all four intact beta-blockers could be detected both in the roots and the leafs of the cress plants. For the investigations on the metabolization of the four beta-blockers, literature was checked for possible phase I metabolites, sometimes also called transformation products (TPs). Focusing on metabolization in mammals, primarily products resulting from oxidation, hydroxylation, and dealkylation were found—this is subsequently mirrored in the aquatic environment where, following excretion, these substances are transported (Iancu et al. 2019; Stankiewicz et al. 2015). In the case of ATE and MET, Koba et al. (2016) (tentatively) identified the following TPs in soil: ATE acid (C_14_H_21_NO_4_), A1 (C_13_H_19_NO_4_), A2 (C_11_H_15_NO_4_), and MET acid (C_14_H_21_NO_4_). In a study conducted by Barclay et al. (2012), two further MET metabolites, namely hydroxylated MET (MET-OH, C_15_H_25_NO_4_) and deaminated MET (C_12_H_16_O_5_), were detected in wastewater. Regarding PRO, three phase I metabolites were found in a critical review by Kasprzyk-Hordern (2010) on pharmacologically active compounds in the environment: hydroxylated PRO (C_16_H_21_NO_3_), dealkylated PRO (C_13_H_14_NO_2_), and naphtaloxylactic acid (C_13_H_11_O_4_).

**Fig. 2:**
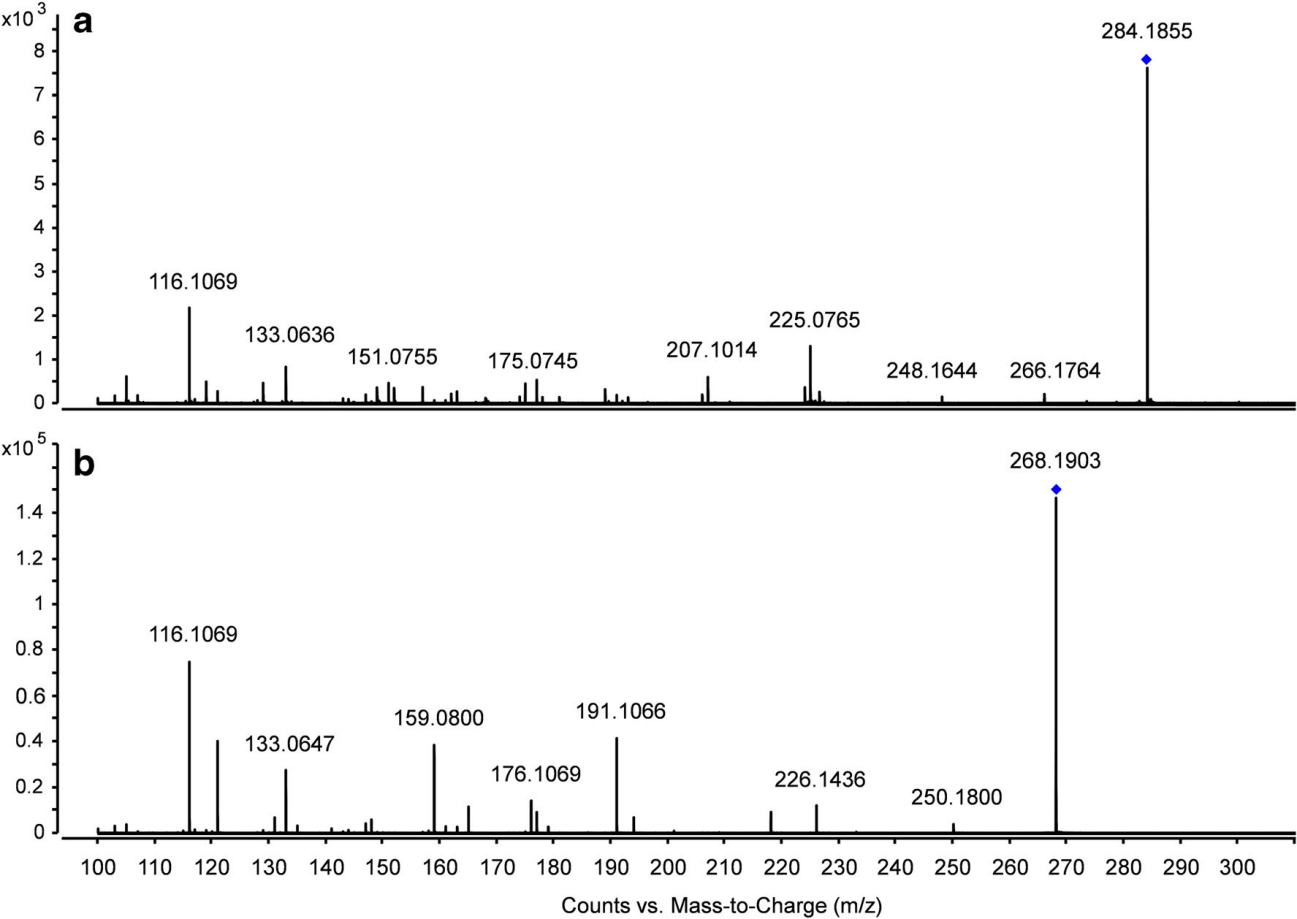
MS/MS spectra of hydroxylated MET (A) and MET (B) in cress sample on day 8 fragmented with a collision energy of 20 V

Based on the so far known phase I metabolites and the general knowledge of possible phase II conjugates, like glucose, malonic acid, glucuronic acid, or glutathione, a database was created within the present work containing several possible combinations and all the plant extracts were checked for these. Despite that, only one phase I metabolite could be detected. In the case of MET, a hydroxylated form (MET-OH, see Fig. 2) apart from the parent drug was tentatively identified. No further phase I or phase II metabolites of the respective pharmaceuticals could be detected. This was quite surprising because, in other studies with cress as a model, phase I and II metabolites indeed could be identified, like in the case of nonsteroidal anti-inflammatory drugs (NSAIDs) or lipid-lowering drugs (Emhofer et al. 2017; Emhofer et al. 2019).

**Fig. 3:**
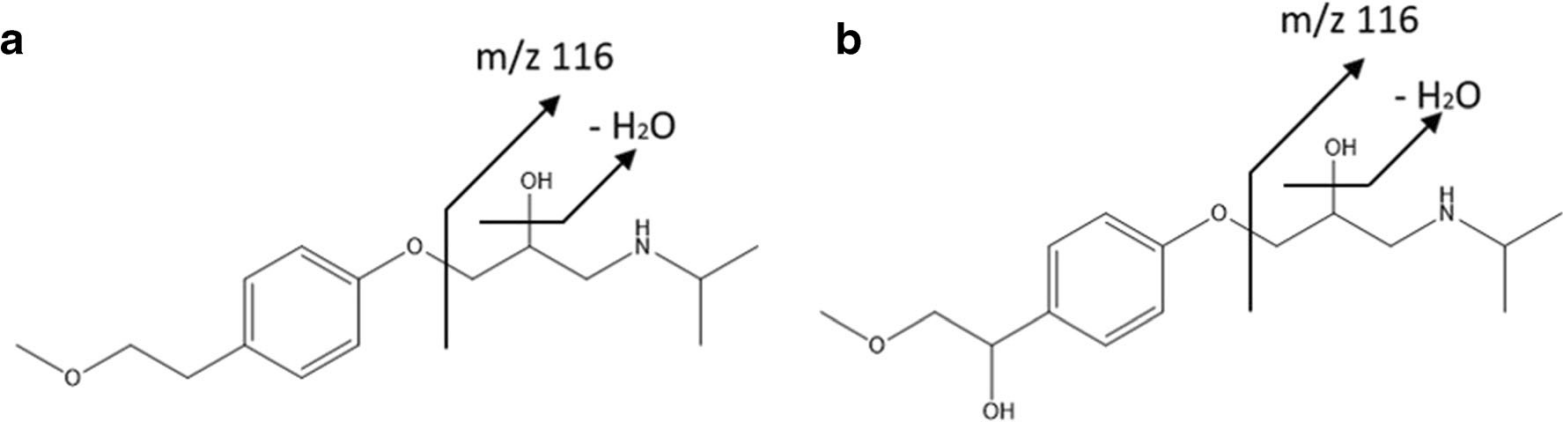
Structure of MET (A) and the proposed structure of hydroxylated MET (B) with their characteristic fragments

The first hint of a probable existence of a hydroxylated MET is the 0.3-min shorter retention time compared to MET due to the slightly higher polarity of the additional hydroxyl group. The tentative identification of MET-OH was based on the comparison to the known MET fragments (see Fig. 2). In the following, the sum formulas of the parent compounds and their respective fragments are from the neutral species, whereas the m/z is given from the [M + H]^+^ species. MET (268.1902 m/z, C_15_H_25_NO_3_) shows the following characteristic fragments: m/z 250.1793 (C_15_H_23_NO_2_, loss of water), m/z 191.1066 (C_12_H_14_O_2_), m/z 159.0801 (C_11_H_10_O), m/z 133.0643 (C_9_H_8_O), and m/z 116.1069 (C_6_H_13_NO). Hydroxylated MET (284.1859 m/z, C_15_H_25_NO_4_) showed the following characteristic fragments: m/z 266.1708 (C_15_H_23_NO_3_), m/z 207.1014 (C_12_H_14_O_3_), m/z 175.0763 (C_11_H_10_O_2_), m/z 133.0653 (C_9_H_8_O), and m/z 116.1073 (C_6_H_13_NO). Both species have a fragment caused by the loss of water and show a characteristic fragment of 116 m/z after cleaving of the phenol derivate. Proposing the added hydroxyl group of the MET-OH on the phenol derivate (see Fig. 3), a mass difference of 16 Da occurs caused by the additional oxygen, which is represented by the two fragments of 191 m/z (MET) and 207 m/z (MET-OH). The same applies for m/z 159 (MET) and m/z 175 (MET-OH).

As MET-OH was also present in the irrigation water, it was suspected that the hydroxylation is caused by environmental reasons like UV-light and oxygen and was not a phase I metabolite produced by the plant. As already mentioned, there was no standard available for a full identification of MET-OH, and therefore, the uptake of MET-OH over time by the cress plant was not analyzed, as a quantification would not have been possible.

### Time study

For the time study, a matrix-matched calibration with the addition of DCF as ISTD was employed. Focusing on the LOQs of the four beta-blockers, a value of 0.5 mg L^−1^ referring to 0.2 mg kg^−1^ of root material or 0.15 mg kg^−1^ of leaves was determined. In addition, matrix effects were investigated by spiking solutions of plant matrix (root and leaves) with appropriate amounts of ATE, PRO, BIS, and MET. Ionization suppression was minimal for the root matrix as at least 90% of the signal area achieved in pure solutions was obtained in every case. The situation was somewhat different for the leaves where suppression effects between 31 and 67% had to be encountered. As can be seen from the data below, relative standard deviations (RSDs) for the drugs taken up by the plant were ranging from less than 10 (PRO in roots) to almost 60% (PRO in shoot)—all related to the measurement of three independent plant samples. It must be taken into consideration that these RSD values, to a substantial degree, are due to biological inhomogeneity and only partly reflect the quality of the analytical method employed.

**Fig. 4:**
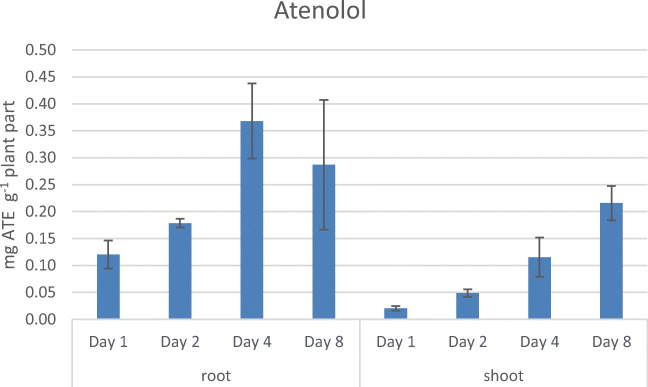
Uptake of ATE over 8 days in *Lepidium sativum* (means from three individually grown plant batches)

During the 8 days of drug exposure, all four beta-blockers were taken up by *Lepidium sativum*. The uptake of ATE increased constantly (with the following amounts given per gram of fresh plant material), starting with 120 μg ± 26 μg ATE per gram wet weight (p. g. w. w.) in the root part, going up to 368 μg ± 70 μg ATE (p. g. w. w.) after day 4, and slightly decreasing to 287 μg ± 120 μg ATE (p. g. w. w.) on day 8. Also, a transfer of the drug from the root part of the cress to the shoots could be observed over this time period, beginning with 21 μg ± 4 μg ATE (p. g. w. w.) at day 1, going up to 216 μg ± 32 μg ATE (p. g. w. w.) on day 8 (see Fig. 4). The relatively low log *P* of ATE of 0.33 might be the reason for the slow but steady uptake in the plant.

**Fig. 5:**
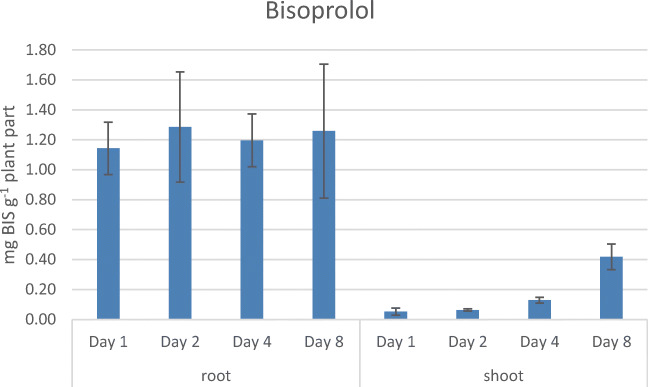
Uptake of BIS over 8 days in *Lepidium sativum* (means from three individually grown plant batches)

In the case of BIS, already at day 1 after the exposure, a quite high amount of 1.14 mg ± 0.17 mg BIS (p. g. w. w.) in the root could be detected. This amount stayed then quite steady over the remaining 7 days, resulting in 1.26 mg ± 0.45 mg BIS (p. g. w. w.) at day 8. In the shoots, a steady progression over the 8 days could be observed: from 0.05 mg ± 0.02 mg BIS (p. g. w. w.) up to 0.42 mg ± 0.09 mg BIS (p. g. w. w.) (see Fig. 5). The high amount of BIS already after day 1 of the treatment seems to be related to the significantly higher log *P* of 1.9 compared to the low log *P* of ATE and therefore, the concentration stayed more or less constant over the time period.

**Fig. 6:**
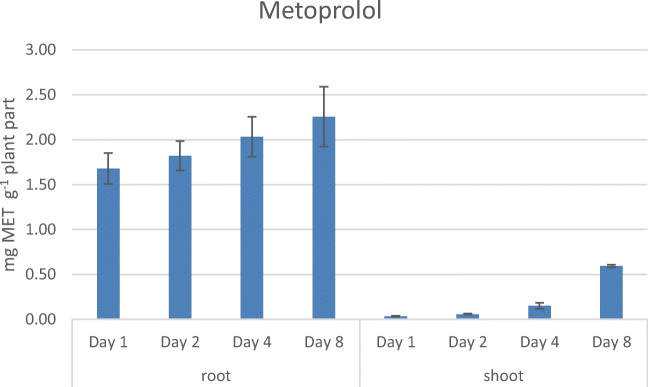
Uptake of MET over 8 days in *Lepidium sativum* (means from three individually grown plant batches)

The amount of MET at day 1 was 1.68 mg ± 0.17 mg MET (p. g. w. w.) in the roots and increased up to 2.26 mg ± 0.33 mg MET (p. g. w. w.) at day 8. In the shoots, 0.04 mg ± 0.01 mg MET (p. g. w. w.) could be determined at day 1, and a concentration of 0.59 mg ± 0.02 mg MET (p. g. w. w.) was reached on day 8 (see Fig. 6).

**Fig. 7:**
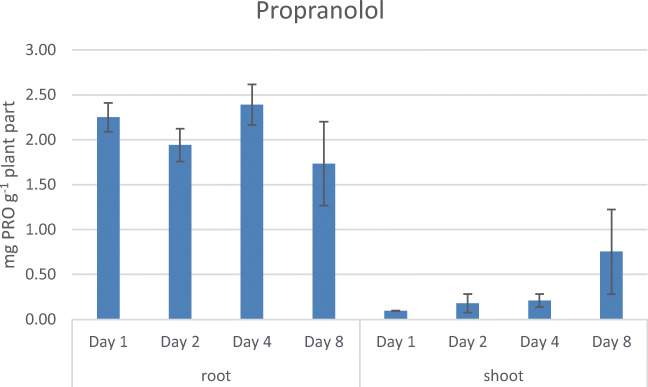
Uptake of PRO over 8 days in *Lepidium sativum* (means from three individually grown plant batches)

Looking at PRO at day 1, 2.25 mg ± 0.16 mg PRO (p. g. w. w.) was taken up by the roots. The PRO concentration stayed quite constant over the remaining 7 days, with concentrations around 2 mg PRO (p. g. w. w.) in the roots. The amount of PRO in the shoots was low over the first 4 days, between 0.10 mg PRO and 0.21 mg PRO (p. g. w. w.), but increased over the remaining 4 days up to 0.75 mg ± 0.47 mg PRO (p. g. w. w.) (see Fig. 7). PRO with the highest log *P* of 2.9 resulted also in the highest drug concentration at day 1 compared to the remaining three beta-blockers.

## Conclusions

In this study, it was revealed that ATE, BIS, MET, and PRO are taken up by cress, but are not metabolized in the plant. Furthermore, it seemed that the log *P* has an influence on the uptake speed of a drug, as ATE with a log *P* of 0.33 was taken up slowly over the course of eight days and the remaining three beta-blockers (BIS, MET, PRO), all with a log *P* > 1.6, were taken up by the roots in high amounts already at day 1 after the exposure and remained constant or increased only slightly over the remaining days. In the case of the translocation of the beta-blockers from the roots to the shoots, the log *P* seems to have no impact, as there was a slow increase of the drug concentration over the whole 8 days for all 4 beta-blockers. In the case of MET, a phase I metabolite, namely hydroxylated MET, could be tentatively identified, but was already present in the irrigation water after 1 week and thus not a result of a metabolization by the plant. It is worth to mention that significant bioaccumulation occurred and that the respective beta-blockers were taken up by the plants in its active form. This on the one hand can lead to a continuous uptake of low amounts of several beta-blockers. On the other hand, although only traces of the various intact beta-blockers could be found in the plants, the mixture of these beta-blockers could have a significant impact on the health of people.

## Data Availability

All data and materials are included in this published article.
